# Preoperative predictors of enucleation time during en bloc ‘no-touch’ holmium laser enucleation of the prostate

**DOI:** 10.1186/s12894-020-00758-4

**Published:** 2020-11-11

**Authors:** Chun-Hsuan Lin, Wen-Jeng Wu, Ching-Chia Li, Sheng-Chen Wen

**Affiliations:** 1 Department of Urology, Kaohsiung Medical University Hospital, Kaohsiung Medical University, No. 100, Tzyou 1st Road, Kaohsiung, 807 Taiwan; 2 Department of Urology, School of Medicine, College of Medicine, Kaohsiung Medical University, Kaohsiung, Taiwan; 3 Graduate Institute of Clinical Medicine, College of Medicine, Kaohsiung Medical University, Kaohsiung, Taiwan

**Keywords:** Holmium laser enucleation of the prostate, Enucleation time, Benign prostatic hyperplasia, En bloc ‘no-touch’ enucleation, Preoperative predictors

## Abstract

**Background:**

To evaluate preoperative predictors of enucleation time during en bloc ‘no-touch’ holmium laser enucleation of the prostate (HoLEP)

**Methods:**

We enrolled 135 patients with symptomatic benign prostatic hyperplasia (BPH) treated with en bloc ‘no-touch’ HoLEP from July 2017 to March 2019 by a single surgeon. Preoperative, perioperative, and postoperative clinical variables were examined. Stepwise linear regression was performed to determine clinical variables associated with enucleation times.

**Result:**

The average (range) enucleation time was 46.1 (12–220) minutes, and the overall operation time was 71 (18–250) minutes. History of antiplatelet agents, history of urinary tract infection (UTI), and increasing specimen weight were each significantly associated with increasing enucleation time. No category IV complications were recorded, and all complications were evenly distributed among the groups according to the HoLEP specimen weight.

**Conclusion:**

En bloc ‘no-touch’ HoLEP was found to be an efficient and reproducible surgical method for treating BPH. Prostatic gland size was significantly associated with increased enucleation times. Similarly, history of UTI and antiplatelet agents were correlated with increased operative time.

## Background

Since the first clinical report on holmium laser enucleation of the prostate (HoLEP) by Gilling et al. [1], multiple randomized controlled trials have been conducted. In many of those trials, compared with open prostatectomy and transurethral resection of the prostate (TURP), HoLEP has been proven to have advantages in size independence and minimal invasiveness for treatment of obstructive symptoms from benign prostatic hyperplasia (BPH), with excellent long-term results [2–6]. A recent study revealed that the cost to inpatients was lower for HoLEP than for open prostatectomy [7]. Despite having been introduced into clinical operative practice two decades ago, the HoLEP technique is still not as widely applied as it deserves given its proven advantages (low morbidity, minimal invasiveness, size independence, long-term durability) [8–10]. Because of the potentially long operative times and steep learning curve, the first frustrating attempts often deter many endo-urologists from continuing to use this method [11].

The initial description of the HoLEP technique has been repeatedly modified over the past 20 years. More recent studies of en bloc procedures could prove the advantages of improving efficiency of enucleation, better visualization on the correct plane, and optimal safety to preserve the sphincter compared to the three-lobe method [18–20]. We started using a holmium laser to reproduce the en bloc ‘no-touch’ technique reported by Scoffone [18] in enucleation of the prostate at our department in 2015. Performance and efficiency of HoLEP relies on the most critical step: transitional zone enucleation. Enucleation time and efficiency depend on several critical factors, such as tissue quality and prostatic volume. Because of the potentially prolonged operative times and arduous learning curve of en bloc HoLEP, the study aim was to evaluate a time predictive model and identify preoperative predictors of enucleation time during en bloc ‘no-touch’ HoLEP to improve patient selection.

## Methods

### Subjects

Between July 2017 and March 2019, 135 consecutive patients who received en bloc ‘no-touch’ HoLEP by the same experienced surgeon (SCW) were admitted to the Department of Urology, Kaohsiung Medical University Hospital, Kaohsiung, Taiwan. Inclusion criteria were as follows: International Prostate Symptom Score (IPSS) > 8, postvoid residual urine volume (PVR) > 50 mL, maximum urinary flow rate (Q max) < 15 mL/s, or men with BPH that causes acute urinary retention. Exclusion criteria were voiding disorders not associated with BPH or clinical medicine history not recorded.

### Study variables and primary outcome

The following factors were analyzed: pre-HoLEP prostate-specific antigen (PSA), age, history of urinary retention requiring Foley catheter, history of 5-alpha-reductase inhibitor (5ARi) use, history of antiplatelet agents with aspirin which not discontinue prior to surgery, history of recurrent urinary tract infections (UTIs) which was defined as urine culture positive more than 3 times in the 3 most recent months without Foley indwelling, prostate volume measured by transrectal ultrasound (TRUS), TURP treatment, and incidental prostatic malignancy in the HoLEP specimen. Patients with the suspicion of prostate carcinoma were ruled out by prostate biopsy if the PSA value or digital rectal examination results were abnormal. All operations were performed by a single surgeon (SCW). Intraoperatively, overall operative time including applied resectoscope until catheter placed, enucleation time, and morcellation time were documented. Enucleation and Morcellation efficiencies were defined as resected adenoma weight divided by enucleation time and morcellation time respectively. The final pathological HoLEP specimen weight was recorded as measured in operation room before sent for formalin fixation. The clinical perioperative variables were analyzed. The primary outcome was enucleation time.

### Description of the procedure

Prostatic adenoma was enucleated by using the en bloc ‘no-touch’ technique. The equipment used included a 100-W holmium laser (Lumenis, Santa Clara, California), 550-μm end-firing fiber, 26-Fr continuous-flow laser resectoscope (Olympus, Hamburg, Germany), and a morcellator (VersaCut, Lumenis) introduced through the working channel of the Storz nephroscope. The first step was started near the verumontanum by finding the bilateral surgical plane. Then, the surgeon turned laterally and ventrally to make the bilateral plane close to the anterior commissure. The median lobe and the rest of the bilateral lobe were dissected by using a retrograde approach, and then the whole adenoma was lifted. The only remaining connection of the adenoma and prostate capsule was the mucosal strip, which was carefully incised by laser without forceful traction. After meticulous hemostasis by holmium laser was achieved, the prostate adenoma was evacuated by morcellation.

Occasionally, unusually tough and difficult-to-dissect prostatic tissue (termed “beach balls”) may be encountered during enucleation, which may prolong the operative time. A 20-Fr 3-way Foley catheter using normal saline for continuous bladder irrigation was inserted at the end of the surgery. Generally, the irrigation fluid flow was gradually tapered down and terminated the morning after the operation. All patients received perioperative antibiotic treatment. After confirming cessation of hematuria, the Foley catheter was removed.

### Statistical analyses

General data were analyzed by using descriptive statistics. For the present study we divided excised specimen weight and prostate volume into groups (W: < 15 g, 15–50 g, 50–80 g, > 80 g; V: < 50 mm^3^, 50–80 mm^3^, > 80 mm^3^) and performed ANOVA between groups to determine significant differences at *P* < 0.05. A simple linear regression analysis of enucleation efficiency measures was performed, and the specimen weight and prostate volume were recorded separately. To identify potential predictors of enucleation time, we used a *P* value of < 0.2 as our criteria for model inclusion, and backward and forward stepwise linear regression models were constructed. All variables in the analysis were included in the initial stepwise linear regression models, and only variables identified as significant (*P* < 0.2) were included in the final presented multiple linear regression models. Prostate weight of < 15 g is used as a reference then dummy variable regression was used between progressive resected specimen weight category in the final presented multiple linear regression models. A *P* value of < 0.05 was accepted as indicative of statistical significance for the final multiple linear regression models. Analyses were performed by using SPSS version 19.0 (IBM SPSS Statistics for Windows, Version 19.0; IBM Corp., Armonk, NY).

## Results

### Patient characteristics

A total of 135 patients were enrolled in this retrospective study. The clinical preoperative characteristics of our study pool are shown in Table 1. The median age was 71.7 (47–95) years. Of the 135 patients, 21 (16%) patients had a history of 5ARi use. Fourteen (10.3%) patients presented with catheter-dependent urinary retention. Twelve (9%) patients had a history of recurrent UTI. Thirteen (10%) patients were receiving ongoing antiplatelet treatment (Aspirin).

**Table 1:** Characteristic of patients undergoing HoLEP

Characteristic	Data
Total patients, n	135
Age (year), mean (SD)	71.7 (9.3)
History of 5ARi use, n (%)	21 (16)
Requiring Foley catheter at the time of HoLEP, n (%)	14 (10.3)
History of UTI, n (%)	12 (9)
History of anticoagulation, n (%)	13 (10)
Pre-HoLEP PSA (ng/mL), mean (range)	6.2 (0.07–1380)
Previous TURP, n (%)	12 (9)
TRUS-P volume (g), mean (SD)	71.1 (42.8)
< 50 ml, n (%)	51 (37.7)
50–80 ml, n (%)	51 (37.7)
> 80 ml, n (%)	33 (24.4)

*5ARi* 5-alpha-reductase inhibitor, *HoLEP* holmium laser enucleation of the prostate, *PSA* prostate-specific antigen, *SD* standard deviation, *TRUS-P* transrectal ultrasound of the prostate, *UTI* urinary tract infection

### Perioperative data

**Fig. 1:**
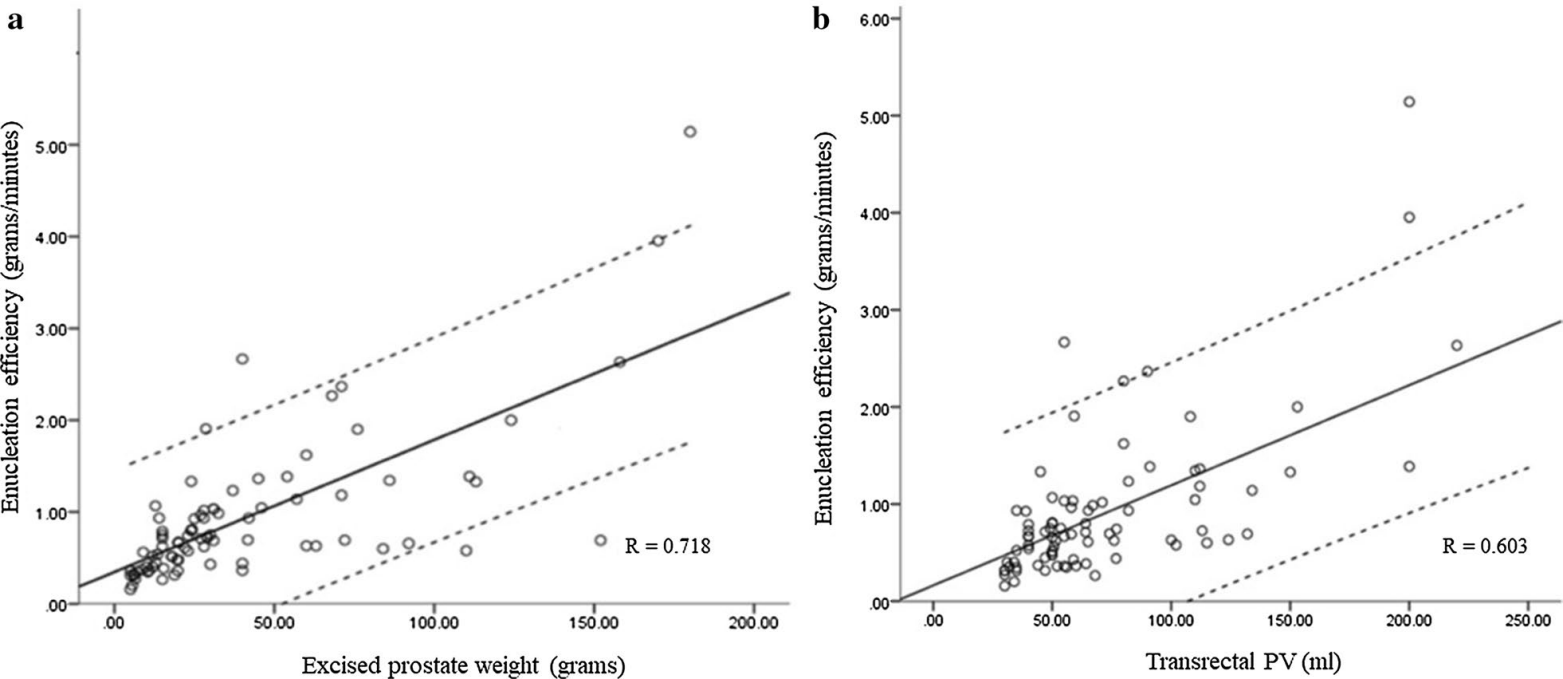
**a** Comparison of enucleation efficiency of HoLEP and specimen weight. **b** Comparison of enucleation efficiency of HoLEP and prostate volume. *The 2 parallel lines was 95% CI

Table 2 shows the perioperative data. The median overall operative time was 71 (18–250) min. The median enucleation and morcellation times were 46.1 (12–220) and 13.3 (4–130) min, respectively. The median enucleation and morcellation efficiencies were 0.9 (0.8) and 4.4 (2.6) g/min, respectively. The overall operative efficiency was 0.5 (0.3) g/min. The advantage of the en bloc ‘no-touch’ technique was especially obvious in large excised adenoma weight that enucleation efficiency increases with large specimen weight (see Fig. 1). Correspondingly, a coherent correlation between prostate volume on TRUS and operation efficiencies was observed.[Pearson’s correlation coefficient (R) for excised prostate weight = 0.718; R for transrectal PV = 0.603].

**Table 2:** Enucleation-associated variables

Variable	Value
Enucleation time (min), mean (range)	46.1 (12–220)
Morcellation time (min), mean (range)	13.3 (4–130)
Overall operation time (min), mean (range)	71 (18–250)
Enucleation efficiency (g/min), mean (SD)	0.9 (0.8)
Morcellation efficiency (g/min), mean (SD)	4.4 (2.6)
Overall operation efficiency (g/min), mean (SD)	0.5 (0.3)
HoLEP specimen weight (g)	
< 15 g, n (%)	38 (28.2)
15–50 g, n (%)	63 (47)
50–80 g, n (%)	16 (11.7)
> 80 g, n (%)	18 (12.9)
Beach ball identified, n (%)	19 (14)
Presence of prostate cancer, n (%)	9 (6.6)

### Enucleation time

To further predict the enucleation time, we analyzed the factors identified as correlating with enucleation time from the stepwise linear regression models, which were HoLEP specimen weight, history of antiplatelet agents, and history of UTI (Table 3). In the final model, history of antiplatelet agents was associated with a 19-min increase in enucleation time (*P* = 0.021). History of UTI was associated with an estimated 24-min increase in enucleation time (*P* = 0.023). Each progressive resected specimen weight category had obvious increases in enucleation time ranging from 17 to 80 min relative to the enucleation time for a specimen weight of < 15 g (Table 3).

**Table 3:** Predictors of enucleation time from multiple linear regression model

Characteristic	Coefficient (min)	95% CI	*P* value
History of UTI	24.23	2.48–45.97	.023
History of antiplatelet agents	19.51	− 3.16 to 42.19	.021
HoLEP specimen weight			
< 15 g	Reference		
15–50 g	17.28	1.01–33.56	.024
50–80 g	36.37	8.33–64.4	.012
> 80 g	80.97	50.66–111.29	< .001
Constant	18.34	4.25–32.43	.011

Other abbreviations as in Table 1. Interpretation of linear regression model: for a patient with a history of UTI and antiplatelet agents, who had a HoLEP specimen weight of 35 g, the estimated enucleation time is 79.36 min (24.23 + 19.51 + 17.28 + 18.34 = 79.36)

*CI* confidence interval

### Complications

Table 4 presents detailed information on all treatment modalities and complications that developed during the first 30 postoperative days. Clavien grades 1 and 2 complications developed in eight patients [Clavien grade 1, 11 (8.1%) patients; Clavien grade 2, three (2.2%) patients] including urinary retention after catheter removal (n = 2), clot retention (n = 9), and postoperative hematuria requiring blood transfusion (n = 3). Clavien grade 3b complications developed in one (0.7%) patient who presented with prostate fossa secondary hemorrhage after HoLEP and needed bipolar coagulation.

**Table 4:** Detailed analysis of Clavien grade 1–3b complication within the 30-day perioperative period

Complication	Treatment	HoLEP (n = 135)
Clavien grade 1 (n = 11; 8.1%)		
Urinary retention after catheter removal	Bedside recatheterization	2 (1.5)
Clot retention without surgical revision	Tamponade evacuation through catheter	9 (6.7)
Clavien grade 2 (n = 3; 2.2%)		
Postoperative hematuria	Transfusion	3 (2.2)
Clavien grade 3b (n = 1; 0.7%)		
Postoperative hemorrhage	Coagulation of prostate fossa	1 (0.7)

## Discussion

BPH is a common disease in aged men that affects quality of life. In the Baltimore Longitudinal Study of Aging, > 60% of men aged ≥ 60 years have some degree of obstructive symptoms caused by BPH [12]. TURP is regarded as the reference standard in the surgical treatment of BPH [13]. Lately, surgical extirpative techniques using lasers, such as holmium and thulium lasers, have been gaining attention as a treatment option for symptomatic BPH. Since Gilling et al. [1] first reported on HoLEP in 1996, it has been proven to be one of the most strictly analyzed surgical treatments for the obstructive symptoms of BPH. More than four randomized controlled trials on this modality have been published [14–17]. More recent descriptions of various approaches to en bloc procedures have been published, mainly to address the goals of improving the effectiveness of enucleation, better visualization on the surgical plane, and optimal safety relative to those of the traditional three-lobe method [18–20]. However, the arduous surgical learning curve and potential long operative times of en bloc HoLEP have been obstacles to its extensive use, despite its obvious advantages. Thus, identifying patient groups and tissue characteristics that may increase operative times may help in appropriate patient selection, proper scheduling of the operating room time, and matching the surgeons’ experience level to the expected difficulty.

Generally, en bloc ‘no-touch’ enucleation involves an “outside-in” procedure that starts at the apex and completely uses a Ho:YAG laser to remove the transitional zone of the prostate. Moreover, the Ho:YAG laser in the vaporization procedure is manipulated as a cutting device. Enucleation time significantly depends on visualization, gland size, and recognition of the dissection plane. Enucleated adenoma weight is predicted to largely affect enucleation times. Several previous studies have reported that the HoLEP operative efficiency increases with larger prostate volumes [21, 23]. In our current series, as expected, regardless of enucleated tissue weight or prostate volume on TRUS, the increase in efficiency was shown by a positive slope on the plots of efficiency versus prostate volume.

Giorgio et al. [24] evaluated the effect of chronic inflammation of the prostate and found that patients with a history of chronic prostatic inflammation have an apparent higher risk of retention. Chronic urinary catheterization and recurrent UTI can hypothetically increase prostate inflammation, which may change the natural morphological architecture, increase gland volume, and obscure the natural plane between the prostate capsule and adenoma. These inflamed prostate tissues may also cause bleeding or oozing during surgery, resulting in poorer visualization and more complicated dissection during en bloc ‘no-touch’ enucleation. In our study, history of UTI history was associated with a 24-min increase in enucleation time (Table 3). However, a Foley in-dwelling catheter at the time of HoLEP was not associated with increased time in the surgical steps of enucleation.

Recent studies have assessed the safety of HoLEP in patients who were taking antiplatelet agents long term and concluded that HoLEP was not a danger to this particular population [25]. This conclusion is expected because the Ho:YAG laser coagulates the bleeding of enucleated tissue with efficiency [8]. We examined whether long-term antiplatelet agents would influence enucleation time and initially hypothesized that because long-term antiplatelet agents could increase bleeding and negatively influence visualization of the operative field, it may increase enucleation time. As expected, our study found that history of long-term chronic antiplatelet agents was related to an apparent increase in enucleation time (Table 3).

Monn. et al. [22] published a retrospective cohort analysis which included a total of 960 patients between 1998 and 2013 illustrating predictor of enucleation and morcellation time during conventional three-lobe HOLEP method. The authors concluded that a history of UTI is associated with an increase in operative time whereas anticoagulation is related to decrease in operative time. The difference impact on the role of antiplatelet agents in surgical time between our present study and the previous published report, in our opinion, is based on difference techniques. The application of en bloc method allows complete adenoma enucleation following surgical capsule at any time, and non-optimal visibility by oozing in patients of long-term antiplatelet agents might lead to increase enucleation time. However, the overall efficiency in the present study (0.5 g/min) indicated no obvious inferior difference compared with earlier randomized clinical trials on the efficiency of retrieval (0.48 g/min) [23]. We believed laminar irrigation between the capsule and enucleated adenoma in en bloc ‘no-touch’ technique help to maintain visualization throughout the procedure compared with chaotic irrigation in the classic 3-lobe method.

The influence of 5ARi use on prostate tissue quality is known to alter the glandular-to-stromal ratio and reduce the volume of overall glandular tissue [26, 27]. For this reason, hypothetically, long-term 5ARi use might increase the prostate fibrous content, which could lead to more difficult enucleation. However, Sandfeldt et al. [28] found that blood loss volume decreased during TURP after using finasteride for 3 months preoperatively. This might decrease bleeding and positively effect visualization during surgery, leading to a faster enucleation rate. Nevertheless, in our study, we found that history of 5ARi use was not actually related to faster enucleation rate. Warner et al. [29] reported the influence of 5ARi use on HoLEP and found that it did not affect HoLEP operative times or outcomes, which is consistent with our study results. In the current study, we found no clear evidence of a relationship between overall HoLEP surgical time and 5ARi use.

We examined whether presence of “beach ball” and previous TURP would impact enucleation operative time. It is believed that beach balls are easy to enucleate relatively. However, multiple beach balls located diffusely in the peripheral edges of adenoma might cause difficult recognition of the dissection plane, and prolong the operative time. The factor of previous TURP might result in hard to identify the correct plane because of natural anatomical structure undermined. We assumed that each factor mentioned above might have a potential role in the prostatic tissue histological architecture and natural plane. Interestingly, neither previous TURP nor presence of beach ball during surgery had a notable effect on enucleation efficiency. Identification of factors associated with development of these difficult prostate tissues is worth studying in the future. We speculate that because of anticipated concerns regarding the effect of dense tissue or a complicated plane, surgeon's great surgical experience could reduce the effect of difficult recognition of the plane between the capsule and enucleated adenoma on overall operative time.

In the current study, all complications after en bloc ‘no-touch’ HoLEP were evenly distributed among the groups according to the HoLEP specimen weight. A prospective larger randomized trial of 61 men with prostate sizes of 40–200 g was reported by Tan et al. [14] in which 30 and 31 patients underwent TURP had HoLEP, respectively. This randomized trial reported that mean Foley in-dwelling time and hospital stay were shorter in HoLEP than in TURP. Outcomes and complication rates were similar in both procedures. The above study supports the statement of Kuntz et al. [30] that HoLEP voiding improvement and perioperative morbidity are not based on prostate gland size. There were rather high range of enucleation time(up to 220 min) and morcellation time (up to 130 min) been noted in the present study. Although relative longer operation time, we didn’t divided procedure into stages or delayed morcellation, since we believe that this will ultimately accelerate patient recovery. No elevated urethral stricture rate in long operation time group of en bloc ‘no-touch’ HoLEP. At the time of the study, our en bloc ‘no-touch’ HoLEP method had been applied for only 1.5 years. Consequently, no long-term follow up data were practicable for interpretation.

This study had some limitations. First, because of its retrospective design, it was intrinsically limited despite inclusion of consecutive patients to avert potential selection bias. Subsequently, we did not consider whether energy usage changed according to the different patient characteristics, but enucleation efficiency may vary depending on the amount of laser energy used. Kim et al. [31] reported a new parameter combining enucleation time and energy consumption to estimate enucleation skills of the operators. It demonstrate that energy consumption decreases as the enucleation technique of a surgeon develops. This trend suggests that as the surgeon’s surgical enucleation skill progresses, less energy is used and efficiency is increased. Lastly, surgeries in the study patient group were performed by a single surgeon. Therefore, we recommend that this enucleation time prediction model of en bloc ‘no-touch’ HoLEP should be examined by multiple surgeons hereafter to determine if it is generally reproducible and acceptable. Despite these limitations, this study examined how preoperative characteristics may affect enucleation times in patients undergoing en bloc ‘no-touch’ HoLEP for BPH and provided a possible enucleation time prediction model. Additionally, the study also found that prostate gland size was not associated with increases in complications after HoLEP.

## Conclusion

This study showed that the operation time of this technique depends on patient characteristic and prostate size. Aside from adenoma size, a history of UTI and antiplatelet agents were associated with an increase in operative time. This useful enucleation time prediction model and significant information could allow surgeons to schedule suitable surgical times for use of an operating room, choose patients based on their characteristics who are most suitable for the procedure, and match a surgeon’s level of experience with the expected degree of surgical difficulty and operative time.

## Supplementary information

**Additional file 1:** Efficacy of holmium laser enucleation of the prostate in patients with a small prostate (≤30 mL).

## Data Availability

Data will be available by contacting the corresponding author. All strains and reagents used in the studies are available upon request.

## Abbreviations

BPH: Benign prostatic hyperplasia
PSA: Prostate-specific antigen
TRUS: Transrectal ultrasound
TURP: Transurethral resection of the prostate
UTI: Urinary tract infection
5ARi: 5-Alpha-reductase inhibitor
HoLEP: Holmium laser enucleation of the prostate
IPSS: International Prostate Symptom Score
PVR: Postvoid residual urine volume
Qmax: Maximum urinary flow rate
PV: Prostate volume
