# Supplementary material for: Preoperative predictors of enucleation time during en bloc ‘no-touch’ holmium laser enucleation of the prostate
Source: BMC Urol. 2020 Nov 11;20:185. doi: 10.1186/s12894-020-00758-4 (PMC7661272; doi:10.1186/s12894-020-00758-4)
Supplement: Supplementary file 1 — Additional file 1: Efficacy of holmium laser enucleation of the prostate in patients with a small prostate (≤30 mL). [file 12894_2020_758_MOESM1_ESM.pdf]

pISSN: 2287-4208 / eISSN: 2287-4690
World J Mens Health 2017 December 35(3): 163-169
https://doi.org/10.5534/wjmh.17011

**Original Article**

# Efficacy of Holmium Laser Enucleation of the Prostate in Patients with a Small Prostate (≤30 mL)

**Sejun Park, Taekmin Kwon, Sungchan Park, Kyung Hyun Moon**

*Department of Urology, Ulsan University Hospital, University of Ulsan College of Medicine, Ulsan, Korea*

**Purpose:** We investigated the effects of holmium laser enucleation of the prostate (HoLEP) in patients with a small prostate (≤30 mL) in whom medical treatment was ineffective.

**Materials and Methods:** Data from 132 patients who underwent HoLEP by a single surgeon between 2012 and 2015 were retrospectively analyzed. All patients received benign prostatic hyperplasia medication for at least 6 months before surgery. The patients were divided into 2 groups according to prostate size: group 1, ≤30 mL (n=30); and group 2, >30 mL (n=102). Clinical characteristics and the International Prostate Symptom Score (IPSS), including quality of life (QoL), peak urinary flow rate (Qmax), and postvoid residual urine (PVR), before surgery and 3 months postoperatively, were compared between the 2 groups.

**Results:** In group 1, the IPSS, QoL, and PVR significantly decreased and the Qmax significantly increased 3 months after surgery. The voiding subscore also significantly decreased 3 months after surgery ($p<0.001$), but the storage subscore was not significantly different ($p=0.055$). In group 2, hemoglobin, the IPSS, QoL, and PVR significantly decreased and the Qmax significantly increased 3 months after surgery. In these patients, both the storage subscore and voiding subscore significantly decreased after surgery (both $p<0.001$). There were no significant differences between the groups in hemoglobin, IPSS, QoL, Qmax, and PVR either before or 3 months after surgery.

**Conclusions:** When other medical treatments are ineffective, HoLEP is an effective intervention for patients with a small prostate.

**Key Words:** Holmium; Lasers; Prostate; Transurethral resection of prostate; Treatment outcome

## INTRODUCTION

Over the past 3 decades, the initial strategy for the treatment of benign prostatic hyperplasia (BPH) has been medical treatment [1]. However, surgery should be considered if lower urinary tract symptoms do not respond to medical treatment over a sufficient treatment period or if patients have complications, such as recurrent urinary retention, gross hematuria, and bladder stone [2,3]. Although transurethral resection of the prostate (TURP) is considered the gold standard for surgical treatment, modern surgical treatments have shifted toward laser procedures and outpatient approaches, with holmium laser enucleation of the prostate (HoLEP) among the treatments now recom-

Received: Mar 23, 2017; Revised: Aug 14, 2017; Accepted: Aug 23, 2017; Published online Nov 20, 2017

Correspondence to: Kyung Hyun Moon https://orcid.org/0000-0002-5966-0025
Department of Urology, Ulsan University Hospital, University of Ulsan College of Medicine, 877 Bangeojinsunhwan-doro, Dong-gu, Ulsan 44033, Korea.
Tel: +82-52-250-7190, Fax: +82-52-250-7198, E-mail: urofirst@hanmail.net

Copyright © 2017 Korean Society for Sexual Medicine and Andrology
This is an Open Access article distributed under the terms of the Creative Commons Attribution Non-Commercial License (http://creativecommons.org/licenses/by-nc/4.0) which permits unrestricted non-commercial use, distribution, and reproduction in any medium, provided the original work is properly cited.

mended by the American Urological Association [4,5]. HoLEP, first introduced in 1995, is effective for the treatment of patients with symptomatic BPH. HoLEP is well-known for its effective treatment of large prostates with no size limitations, and allows complete enucleation of the transitional zone, mimicking open prostatectomy but with much less morbidity [6,7].

Prostate size is an important factor to consider when treatments for benign prostate obstruction (BPO) are being evaluated [8]. In the medical treatment era, a small prostate may be initially treated with medical therapy [1]. Clinicians might be hesitant to perform surgical treatment and prefer to continue medical treatment, even if surgery is indicated, in patients with a small prostate. In these patients, the interval between medical treatment and surgery can increase and they may not be able to undergo surgery within the appropriate time interval. If patients have bladder decompensation due to delayed surgery, the success rate of the surgery can decrease [3,9].

The impact of prostate size on the outcomes of HoLEP surgery has been extensively studied, and most published data have been from patients with a large prostate [7,8,10]. Some urologists believe that HoLEP is only appropriate for large prostates. Only a few studies have addressed small prostates, and surgeons may be reluctant to operate on such prostates [11,12]. Moreover, because the cutoff value for a small prostate in these studies was 40 to 60 mL [8,12], it remains unclear whether HoLEP is effective in patients with a very small prostate (≤30 mL). Therefore, in our current study, we investigated the effects of HoLEP in patients with a very small prostate (≤30 mL) when other medical treatment was ineffective.

## MATERIALS AND METHODS

### 1. Ethics statement

This research was performed with the agreement and supervision of the Institutional Review Board of Ulsan University Hospital, and the requirement for informed consent was waived because of the retrospective design of the study.

### 2. Study participants

The records of 132 patients who underwent HoLEP by a single surgeon between 2012 and 2015 were reviewed. All patients received BPH medication for at least 6 months before surgery. Patient characteristics, including age, body mass index, hemoglobin level, prostate-specific antigen level, prostate size on transrectal ultrasonography (TRUS), enucleation weight, and enucleation time were collected. The International Prostate Symptom Score (IPSS), including the quality of life score (QoL), peak urinary flow rate (Qmax), and postvoid residual urine (PVR), were evaluated before surgery and 3 months after. The IPSS was divided into 2 subgroups, corresponding to the voiding scores and storage scores. Cystoscopy was performed to examine obstruction and bladder trabeculation in all patients before HoLEP, and the trabeculation grade was classified based on a previous study [13]. Patients were excluded if they had been previously diagnosed with neurogenic bladder, prostate cancer, or urethral stricture or had undergone a previous surgical intervention related to BPH. The patients were divided into 2 groups according to their prostate volume on TRUS: group 1, prostate volume ≤30 mL (n=30); and group 2, prostate volume >30 mL (n=102).

### 3. Intervention

All prostatic adenomas were enucleated by the 2- or 3-lobe technique. The tools used for the HoLEP procedure included a 26-Fr continuous flow laser resectoscope, a laser-fiber stabilizing bridge, a 100-w holmium laser (VersaPulse; Lumenis Ltd., Yokneam, Israel), and a 550-μm end-firing laser fibers (SlimLine; Lumenis Ltd.). A 26-Fr nephroscope and a tissue morcellator (Versacut; Lumenis Ltd.) were used to remove enucleated tissue. The enucleated tissues were immediately weighed and examined histologically. After surgery, a 3-way 22-Fr Foley catheter was inserted with continuous bladder irrigation and removed 1 to 2 days after surgery.

### 4. Outcome measures

Changes in objective (Qmax and PVR) and subjective (IPSS and QoL) urinary parameters were assessed before surgery and 3 months after. Operative time, hospitalization period, catheterization period, and postoperative complications were reported, graded using the modified Clavien scale, and compared between the study groups [14,15].

## 5. Statistical analysis

The clinical characteristics of the 2 groups were compared with the Student t-test for continuous variables and with the Pearsons chi-square test for categorical variables. The paired t-test was used to assess the changes in continuous measures between before surgery and 3 months after. Quantitative data are expressed as mean values with standard deviations or as median values with ranges. Hemoglobin, the IPSS, QoL, Qmax, and PVR were compared between before surgery and 3 months after in each patient group. The changes in hemoglobin, the IPSS, QoL, Qmax, PVR, enucleated tissue weight, and enucleated time were compared between the groups. All statistical tests were 2-sided, with $p<0.05$ considered significant. Data were analyzed using IBM SPSS Statistics ver. 21 (IBM Co., Armonk, NY, USA).

# RESULTS

The mean age of the entire patient group was 66.1 years, and all patients showed lower urinary tract symptoms that failed to respond to medication. Of the 132 patients, 12 (9.1%) had recurrent urinary retention and 3 (2.3%) had bladder stones. In the cystoscopy findings of the 30 patients in group 1, all patients had BPO, and 4 patients had a high bladder neck with posterior commissural hyperplasia. Bladder trabeculation was grade 0 in 4 patients (13.3%), grade 1 in 23 patients (76.7%), grade 2 in 2 patients (6.7%), and grade 3 in 1 patient (3.3%). The patients in group 2 had a higher grade of trabeculation than those in group 1 ($p=0.031$). Descriptive characteristics according to group are presented in Table 1. Compared with the patients in group 2, the patients in group 1 had a lower level of prostate-specific antigen (3.1±2.4 ng/mL *vs.* 1.5±1.6 ng/mL, $p=0.016$). There were no differences between the 2 groups in any other variables, including age, body mass index, the IPSS, QoL, Qmax, and PVR.

Table 2 shows the perioperative and postoperative data of each group. In group 1, the IPSS, QoL, and PVR significantly decreased (19.3±8.7 *vs.* 9.1±5.6, $p<0.001$; 3.9±1.3 *vs.* 1.8±0.9, $p<0.001$; 57.3±70.7 mL *vs.* 20.5±22.2 mL, $p=0.032$) and the Qmax significantly increased (9.9±4.3 mL/s *vs.* 16.9±7.9 mL/s, $p<0.001$) 3 months after surgery. Three months after surgery, the voiding subscore had also decreased (12.6±6.1 *vs.* 3.9±3.5, $p<0.001$) but the storage subscore was not different (6.9±4.2 *vs.* 5.1±2.6, $p=0.055$). Three months after sur-

**Table 1.** Patients' characteristics and preoperative data

| Variable | Group 1[a] | Group 2[a] | p-value |
|---|---|---|---|
| Mean age (y) | 66.2±9.4 (66.5, 55∼80) | 66.1±8.1 (65.5, 46∼82) | 0.274 |
| Mean body mass index (kg/m$^2$) | 24.4±2.6 (24.1, 16.7∼31.5) | 24.4±2.4 (24.5, 18.2∼33.1) | 0.986 |
| Mean PSA (ng/mL) | 1.5±1.6 (0.9, 0.1∼7.2) | 3.1±2.4 (2.4. 0.3∼9.8) | 0.016 |
| Mean Hb (ng/mL) | 14.3±1.6 (14.1, 10.9∼16.1) | 14.1±1.1 (13.9, 11.1∼15.9) | 0.647 |
| Mean prostate volume on TRUS (mL) | 24.1±3.4 (24.5, 16.0∼30.0) | 60.0±23.7 (51.6, 30.7∼216) | <0.001 |
| Mean IPSS total | 19.3±8.7 | 17.9±8.0 | 0.506 |
| Voiding subscore | 12.6±6.1 | 10.6±5.4 | 0.152 |
| Storage subscore | 6.9±4.2 | 7.4±3.8 | 0.938 |
| Mean IPSS quality of life | 3.9±1.3 | 3.6±1.4 | 0.501 |
| Mean maximal urinary flow rate (mL/s) | 9.9±4.3 | 10.4±5.2 | 0.716 |
| Postvoid residual urine volume (mL) | 57.3±70.7 | 57.6±70.1 | 0.990 |
| Cystoscopic grade of bladder trabeculation[b] | | | 0.031 |
| 0∼1 | 27 | 71 | |
| 2∼3 | 3 | 31 | |

Values are presented as mean±standard deviation (median, range) or number only.
PSA: prostate-specific antigen, Hb: hemoglobin, TRUS: transrectal ultrasonography, IPSS: International Prostate Symptom Score.
[a]The patients were divided into 2 groups according to their prostate volume on TRUS: group 1, prostate volume ≤30 mL (n=30); and group 2, prostate volume >30 mL (n=102).
[b]Classification according to the standard of the Jung et al's paper [13].

**Table 2.** Perioperative data and postoperative outcomes of the 2 groups

| Variable | Before HoLEP | After HoLEP | p-value |
|---|---|---|---|
| Group 1[a] | | | |
| Hb (g/dL) | 14.3±1.6 | 13.5±1.4 | 0.076 |
| IPSS total | 19.3±8.7 | 9.1±5.6 | <0.001 |
| Voiding subscore | 12.6±6.1 | 3.9±3.5 | <0.001 |
| Storage subscore | 6.9±4.2 | 5.1±2.6 | 0.055 |
| IPSS quality of life | 3.9±1.3 | 1.8±0.9 | <0.001 |
| Maximal urinary flow rate (mL/s) | 9.9±4.3 | 16.9±7.9 | <0.001 |
| Postvoid residual urine volume (mL) | 57.3±70.7 | 20.5±22.2 | 0.032 |
| Group 2[a] | | | |
| Hb (g/dL) | 14.1±1.1 | 12.8±1.4 | <0.001 |
| IPSS total | 17.9±8.0 | 5.6±4.0 | <0.001 |
| Voiding subscore | 10.6±5.4 | 1.6±2.1 | <0.001 |
| Storage subscore | 7.4±3.8 | 4.0±2.9 | <0.001 |
| IPSS quality of life | 3.6±1.4 | 1.6±1.2 | <0.001 |
| Maximal urinary flow rate (mL/s) | 10.4±5.2 | 16.4±8.8 | <0.001 |
| Postvoid residual urine volume (mL) | 57.6±70.1 | 25.7±34.2 | <0.001 |

Values are presented as mean±standard deviation.
HoLEP: holmium laser enucleation of the prostate, Hb: hemoglobin, IPSS: International Prostate Symptom Score.
[a]The patients were divided into 2 groups according to their prostate volume on TRUS: group 1, prostate volume ≤30 mL (n=30); and group 2, prostate volume >30 mL (n=102).

**Table 3.** Comparison of perioperative data and postoperative outcomes according to prostate size

| Variable | Group 1[a] | Group 2[a] | p-value |
|---|---|---|---|
| Hb decrease (g/dL) | 0.8±1.1 | 1.3±1.2 | 0.105 |
| IPSS total improvement | 10.0±7.9 | 12.3±8.2 | 0.308 |
| Voiding subscore improvement | 8.6±6.7 | 8.9±5.6 | 0.826 |
| Storage subscore improvement | 1.8±3.7 | 3.5±4.2 | 0.130 |
| IPSS quality of life improvement | 2.1±1.4 | 2.1±1.6 | 0.968 |
| Maximal urinary flow rate improvement (mL/s) | 7.5±9.1 | 8.9±9.8 | 0.541 |
| Postvoid residual urine volume decrease (mL) | 36.9±75.3 | 31.8±66.4 | 0.772 |
| Enucleated tissue weight (g) | 5.7±4.5 | 20.5±16.5 | <0.001 |
| Enucleated tissue ratio (%)[b] | 22.3±16.8 | 31.7±17.3 | 0.032 |
| Enucleation time (min) | 23.9±15.0 | 48.1±37.1 | 0.040 |
| Complication[c] | 5 (16.7) | 6 (5.9) | 0.124 |
| Grade 1 | 2 (6.7) | 3 (2.9) | |
| Grade 2 | 0 (0) | 1 (1.0) | |
| Grade 3a | 1 (3.3)[d] | 0 (0) | |
| Grade 3b | 2 (6.7)[d] | 2 (2.0)[e] | |

Values are presented as mean±standard deviation or number (%).
Hb: hemoglobin, IPSS: International Prostate Symptom Score.
[a]The patients were divided into 2 groups according to their prostate volume on TRUS: group 1, prostate volume ≤30 mL (n=30); and group 2, prostate volume >30 mL (n=102).
[b]Enucleated tissue weight/total prostate size×100.
[c]Modified Clavien Classification System.
[d]Bladder neck contracture.
[e]Urethral stricture.

gery, hemoglobin, the IPSS, QoL, and PVR had significantly decreased and the Qmax had significantly increased in group 2 ($p<0.001$). In these patients, not only did the storage subscore significantly decrease after surgery ($7.4\pm3.8$ *vs.* $4.0\pm2.9$, $p<0.001$), but so did the voiding subscore ($10.6\pm5.4$ *vs.* $1.6\pm2.1$, $p<0.001$).

There were no significant differences between the groups in the changes in hemoglobin, IPSS, QoL, Qmax, and PVR between before surgery and 3 months after surgery. However, patients in group 1 had a lower enucleated tissue ratio ($31.7\%\pm17.3\%$ *vs.* $22.3\%\pm16.8\%$, $p=0.032$) and shorter enucleation time ($48.1\pm37.1$ min *vs.* $23.8\pm15.0$ min, $p=0.040$) than those in group 2. Although the complication rate was not significantly different between the groups, the patients in group 1 were more likely to have higher-grade complications requiring further procedures ($\geq$ grade 3) (Table 3).

## DISCUSSION

Since HoLEP was first introduced, it has rapidly replaced TURP as the gold standard for the surgical treatment of symptomatic BPO. Thus, HoLEP is now the most commonly used surgical technique for treating this condition. According to several recent studies [10,16], HoLEP can be safely performed on prostates of various sizes with excellent treatment results. However, few studies have been conducted of HoLEP in patients with a small prostate, and the surgical management of BPO secondary to a small prostate has only infrequently been discussed [11,12]. A small prostate that may have been resected decades previously may now be initially treated with medical therapy [9]. Even when surgically indicated, clinicians may be hesitant to perform surgery and prefer to continue medical treatment in patients with a small prostate.

The interval between medical treatment and surgery has increased, and contemporary preoperative prostate size has increased since medical treatment became a first-line therapy for surgical candidates with BPH [9,17]. In 1998, 36% of men undergoing TURP had previously been treated with medical interventions, a rate that increased to 87% by 2008 [18]. These findings show that there has been a significant increase in the prevalence of BPH progression-related complications before surgery. A high failure to void rate was reported after TURP due to detrusor hypocontractility after prolonged BPO caused by an initial prolonged medical treatment [9,18]. The cause of failure to void after TURP might not be the recurrent BPO, but detrusor deterioration due to prolonged medical treatment [19]. Thus, performing surgery before the development of detrusor deterioration is important. In our current study, HoLEP was found to be an effective treatment not only for patients with a large prostate, but also for those with a small prostate. Therefore, when medical treatment is not effective, surgeons should decide to perform surgical treatment without hesitation to decrease the time interval between medical treatment and surgery, providing the patient with the chance to undergo surgery within the appropriate time, regardless of prostate size.

In the present study, we performed cystoscopy to examine the presence of BPO and the grade of trabeculation in all patients before surgery. In the cystoscopy findings of the 30 patients in group 1, all patients had BPO and more than 90% had grade 0 or 1 trabeculation. Only 3 patients in group 1 had grade 2 or 3 trabeculation. After surgery, the IPSS decreased from 20 to 14 in patients with grade 2 trabeculation and from 21 to 15 in patients with grade 3 trabeculation. In contrast, the IPSS decreased from 18 to 8 in patients with grade 0 or 1 trabeculation. Trabeculation is a compensatory response of the bladder to complete the emptying of urine, and reveals impairment of the detrusor muscle [20,21]. In patients with high-grade trabeculation, the timing of surgery might have been late. Based on our experiences, we recommend cystoscopy in BPH patients with a small prostate and patients with persistent lower urinary tract symptoms when medical treatment is not effective. If there is definite BPO and mild trabeculation in the bladder, active early surgical treatment may be more helpful for these patients than continuing medical treatment.

In the current study, in patients with a prostate size $\leq30$ mL, the IPSS voiding subscore significantly decreased 3 months after HoLEP ($12.6\pm6.1$ *vs.* $3.9\pm3.5$, $p<0.001$) but the IPSS storage subscore was not significantly different ($6.9\pm4.2$ *vs.* $5.1\pm2.6$, $p=0.055$). Kang et al [22] reported that the improvement in storage symptoms was not significant after TURP in patients with a prostate size $\leq30$ mL, but was significant in patients with a prostate size $>30$

mL. These findings coincide with our results. In our study, compared with patients with a prostate size ≤30 mL, the rate of improvement in storage symptoms was higher in patients with a prostate size >30 mL (63.6% *vs.* 77.5%). The mechanism underlying the improvement in storage symptoms observed after surgery remains unclear [23]. In addition, the influence of prostate size on the improvement in storage symptoms is poorly understood. However, QoL significantly improved in patients with a small prostate ($p<0.001$), which is in itself sufficient reason to consider surgery in these patients.

The rate of bladder neck contracture (BNC) after transurethral prostate surgery was reported to be 2.7% in a long-term study [24]. BNC is one of the most common complications causing retreatment after surgical management of BPO due to BPH, particularly in patients with a smaller prostate [25,26]. Krambeck et al [27] documented a positive correlation between prostate size and postoperative BNC and routinely performed incision of the bladder neck after HoLEP in patients with a prostate size <40 mL. In the present study, 3 patients (10% of patients with a prostate size ≤30 mL, 2.3% of the entire cohort) had BNC after HoLEP and underwent transurethral incision of the bladder neck. No patients with a prostate size >30 mL developed BNC. These 3 patients underwent HoLEP early during the learning curve of the procedure, which probably explains the complications and indicates that the procedure had not yet been mastered. No major surgical complications were seen in our study series, including bladder injury during morcellation, and no patients had urinary incontinence after HoLEP. The main handicap of HoLEP is its steep learning curve, and HoLEP is known to be technically more difficult for both large and small prostates [28,29]. In small prostates, enucleation requires skill and capsular perforation may occur more frequently. It also seems to be difficult to identify the apical plane for enucleation in small prostates [29,30]. Sufficient surgical experience is necessary to reduce the HoLEP complication rate in patients with a small prostate.

The present study was limited by its retrospective nature, the relatively small number of patients, and the presence of significant differences between patient groups. Furthermore, the follow-up period was relatively short. Nonetheless, our study findings are clinically significant because few previous reports have investigated the impact of HoLEP after medical treatment in patients with a very small prostate. In addition, we recommend preoperative cystoscopy to examine BPO and bladder trabeculation as part of careful patient selection.

# CONCLUSIONS

When medical treatment is not effective, HoLEP is an effective intervention with few complications not only in patients with a large prostate, but also in those with a small prostate. However, because the current cohort was comparatively small, further studies are required to determine the clinical validity of this treatment plan.

## Disclosure

The authors have no potential conflicts of interest to disclose.

## Author Contribution

Research conception & design: Park S, Moon KH. Performing the experiments: Park S, Park S. Data acquisition: Park S, Kwon T. Data analysis and interpretation: Park S, Kwon T. Statistical analysis: Kwon T, Park S. Drafting of the manuscript: Park S, Park S, Moon KH. Critical revision: Park S, Moon KH. Receiving grant: Moon KH. Approval of final manuscript: all authors.

## ORCID

Sejun Park, https://orcid.org/0000-0002-7647-4355
Taekmin Kwon, https://orcid.org/0000-0003-2280-319X
Sungchan Park, https://orcid.org/0000-0002-2337-983X
Kyung Hyun Moon, https://orcid.org/0000-0002-5966-0025

# REFERENCES

1. McConnell JD, Roehrborn CG, Bautista OM, Andriole GL Jr, Dixon CM, Kusek JW, et al. The long-term effect of doxazosin, finasteride, and combination therapy on the clinical progression of benign prostatic hyperplasia. N Engl J Med 2003;349:2387-98.
2. Ruszat R, Wyler S, Forster T, Reich O, Stief CG, Gasser TC, et al. Safety and effectiveness of photoselective vaporization of the prostate (PVP) in patients on ongoing oral anticoagulation. Eur Urol 2007;51:1031-8; discussion 1038-41.
3. Giannarini G. Editorial comment on: Holmium laser enucleation versus transurethral resection of the prostate: 3-year follow-up results of a randomized clinical trial. Eur Urol

2007;52:1463.
4. Malaeb BS, Yu X, McBean AM, Elliott SP. National trends in surgical therapy for benign prostatic hyperplasia in the United States (2000-2008). Urology 2012;79:1111-6.
5. McVary KT, Roehrborn CG, Avins AL, Barry MJ, Bruskewitz RC, Donnell RF, et al. Update on AUA guideline on the management of benign prostatic hyperplasia. J Urol 2011; 185:1793-803.
6. Gilling PJ, Cass CB, Malcolm AR, Fraundorfer MR. Combination holmium and Nd:YAG laser ablation of the prostate: initial clinical experience. J Endourol 1995;9:151-3.
7. Masui K, Yoshimura K, Nishiyama H, Okuno H, Ogawa O. Transition in type of surgery for benign prostatic hyperplasia: a multi-institutional study in Japan. Low Urin Tract Symptoms 2011;3:104-8.
8. Kuntz RM, Lehrich K, Ahyai S. Does perioperative outcome of transurethral holmium laser enucleation of the prostate depend on prostate size? J Endourol 2004;18:183-8.
9. Mayer EK, Kroeze SG, Chopra S, Bottle A, Patel A. Examining the 'gold standard': a comparative critical analysis of three consecutive decades of monopolar transurethral resection of the prostate (TURP) outcomes. BJU Int 2012; 110:1595-601.
10. Yin L, Teng J, Huang CJ, Zhang X, Xu D. Holmium laser enucleation of the prostate versus transurethral resection of the prostate: a systematic review and meta-analysis of randomized controlled trials. J Endourol 2013;27:604-11.
11. Lee MH, Yang HJ, Kim DS, Lee CH, Jeon YS. Holmium laser enucleation of the prostate is effective in the treatment of symptomatic benign prostatic hyperplasia of any size including a small prostate. Korean J Urol 2014;55:737-41.
12. Elshal AM, Elkoushy MA, Elmansy HM, Sampalis J, Elhilali MM. Holmium:YAG transurethral incision versus laser photoselective vaporization for benign prostatic hyperplasia in a small prostate. J Urol 2014;191:148-54.
13. Jung JH, Cho SY, Yoo C, Oh SJ. Establishment of the novel cystoscopic classification for bladder trabeculation of neurogenic bladder. Urology 2014;84:515-9.
14. Ku JH, Kim SW, Paick JS. Impact of prostate volume on the efficacy of high-power potassium-titanyl-phosphate photoselective vaporization of the prostate: a retrospective, short-term follow-up study on evaluating feasibility and safety. Yonsei Med J 2010;51:877-82.
15. Morgan M, Smith N, Thomas K, Murphy DG. Is Clavien the new standard for reporting urological complications? BJU Int 2009;104:434-6.
16. Kuntz RM, Lehrich K, Ahyai SA. Holmium laser enucleation of the prostate versus open prostatectomy for prostates greater than 100 grams: 5-year follow-up results of a randomised clinical trial. Eur Urol 2008;53:160-6.
17. Logie J, Clifford GM, Farmer RD. Incidence, prevalence and management of lower urinary tract symptoms in men in the UK. BJU Int 2005;95:557-62.
18. Izard J, Nickel JC. Impact of medical therapy on transurethral resection of the prostate: two decades of change. BJU Int 2011;108:89-93.
19. Thomas AW, Cannon A, Bartlett E, Ellis-Jones J, Abrams P. The natural history of lower urinary tract dysfunction in men: minimum 10-year urodynamic followup of transurethral resection of prostate for bladder outlet obstruction. J Urol 2005;174:1887-91.
20. Khoury AE, Dave S, Peralta-Del Valle MH, Braga LH, Lorenzo AJ, Bägli D. Severe bladder trabeculation obviates the need for bladder outlet procedures during augmentation cystoplasty in incontinent patients with neurogenic bladder. BJU Int 2008;101:223-6.
21. Holm NR, Horn T, Smedts F, Nordling J, de la Rossette J. The detrusor muscle cell in bladder outlet obstruction: ultrastructural and morphometric findings. Scand J Urol Nephrol 2003;37:309-15.
22. Kang YJ, Kim KH, Seo Y, Lee KS. Effect of transurethral resection of the prostate on storage symptoms in patients with benign prostatic hyperplasia of less than 30 ml. World J Mens Health 2013;31:64-9.
23. Saito K, Hisasue S, Ide H, Aoki H, Muto S, Yamaguchi R, et al. The impact of increased bladder blood flow on storage symptoms after holmium laser enucleation of the prostate. PLoS One 2015;10:e0129111.
24. Ficarra V, Cavalleri S. Editorial comment on: holmium laser enucleation versus transurethral resection of the prostate: 3-year follow-up results of a randomized clinical trial. Eur Urol 2007;52:1464.
25. Reich O, Gratzke C, Bachmann A, Seitz M, Schlenker B, Hermanek P, et al. Morbidity, mortality and early outcome of transurethral resection of the prostate: a prospective multicenter evaluation of 10,654 patients. J Urol 2008;180: 246-9.
26. Ahyai SA, Lehrich K, Kuntz RM. Holmium laser enucleation versus transurethral resection of the prostate: 3-year follow-up results of a randomized clinical trial. Eur Urol 2007; 52:1456-63.
27. Krambeck AE, Handa SE, Lingeman JE. Experience with more than 1,000 holmium laser prostate enucleations for benign prostatic hyperplasia. J Urol 2010;183:1105-9.
28. Kim M, Piao S, Lee HE, Kim SH, Oh SJ. Efficacy and safety of holmium laser enucleation of the prostate for extremely large prostatic adenoma in patients with benign prostatic hyperplasia. Korean J Urol 2015;56:218-26.
29. Haraguchi T, Takenaka A, Yamazaki T, Nakano Y, Miyake H, Tanaka K, et al. The relationship between the reproducibility of holmium laser enucleation of the prostate and prostate size over the learning curve. Prostate Cancer Prostatic Dis 2009;12:281-4.
30. Nam JK, Kim HW, Lee DH, Han JY, Lee JZ, Park SW. Risk factors for transient urinary incontinence after holmium laser enucleation of the prostate. World J Mens Health 2015; 33:88-94.
